# Imaging study of ventricular scar in arrhythmogenic right ventricular cardiomyopathy/dysplasia: comparison of three-dimensional electroanatomic voltage mapping and contrast-enhanced cardiac magnetic resonance

**DOI:** 10.1186/1532-429X-13-S1-P249

**Published:** 2011-02-02

**Authors:** Martina Perazzolo Marra, Loira Leoni, Barbara Bauce, Alessandro Zorzi, Manuel De Lazzari, Francesco Corbetti, Luisa Cacciavillani, Ilaria Rigato, Federico Migliore, Maria Silvano, Cristina Basso, Francesco Tona, Gianfranco Buja, Gaetano Thiene, Sabino Iliceto, Domenico Corrado

**Affiliations:** 1 Department of Cardiac, Thoracic, and Vascular Sciences, University of Padua, Padua, Italy; 2 Department of Radiology, Padua, Italy; 3 Department of Medical-Diagnostic Sciences University of Padua, Padua, Italy

## Introduction

The hallmark lesion of arrhythmogenic right ventricular cardiomyopathy (ARVC/D) is the RV myocardial loss with replacement by fibrofatty tissue. Emerging tools offer the possibility to directly visualize fibrofatty ventricular scar. electroanatomic voltage mapping (EVM) by CARTO system has been demonstrated to identify low-voltage myocardial areas (“electroanatomic scar”, EAS) invasively, whereas contrast-enhanced cardiac magnetic resonance CE-CMR has the potential to detect regions of delayed contrast-enhancement (“DCE scar”) non-invasively.

## Purpose

The aim of the present study was to compare EVM and CE-CMR for imaging scar lesion in ARVC/D patients.

## Methods

23 consecutive patients (16 males and 7 females; mean age 38±12 yrs) with a clinical diagnosis of ARVC/D who additionally underwent both RV EVM and CE-CMR. Analysis of RV free wall regions for scar location included the RV outflow tract (RVOT), the antero-lateral region, the infero-basal region and the apex.

## Results

RV EVM was abnormal in 21/23 (91%) patients, with a total of 45 EAS: 17 (38%) in the infero-basal region, 12 (26.6%) in the antero-lateral region, 8 (17.7%) in the RV outflow tract (RVOT) and 8 (17.7%) in the apex. RV DCE was found in 9/23 (39%) patients with a total of 23 RV DCE scars: 4 (17.4%) in the infero-basal region, 9 (39.1%) in the antero-lateral region, 4 (17.4%) in the RVOT and 6 (26.1%) in the apex. Comparative analysis showed a mismatch in 24 RV scar areas, with 22 EAS not confirmed by the DCE (13 in the infero-basal region, 3 in antero-lateral region, 4 in RVOT and 2 on apex), and 2 DCE scars (both in the RVOT) undetected by the EVM. In 9/12 (75%) patients with abnormal RV EVM/normal RV DCE, ≥ 1 DCE were identified in the left ventricular (LV) free wall or interventricular septum, which affected the subepicardial/midmural layers. Overall, ventricular DCE was detected by CE-CMR in 78% of ARVC/D patients.

## Conclusions

EVM and CE-CMR allow identification of RV scar lesions in the majority of ARVC/D patients, although a mismatch between the two techniques was found with fewer RV scars identified by CE-CMR. The high prevalence of LV DCE in ARVC/D patients with abnormal RV-EVM/normal RV-DCE confirms the frequent biventricular involvement and points out the diagnostic relevance of LV scar detection by CE-CMR.

